# Supplementary material for: Glycopeptidomics Analysis of a Cell Line Model Revealing Pathogenesis and Potential Marker Molecules for the Early Diagnosis of Gastric MALT Lymphoma
Source: Front Cell Infect Microbiol. 2021 Aug 12;11:715454. doi: 10.3389/fcimb.2021.715454 (PMC8407071; doi:10.3389/fcimb.2021.715454)
Supplement: Supplementary file 1 [file DataSheet_1.pdf]

Glycopeptidomics Analysis of a Cell Line Model Revealing Pathogenesis and Potential Marker Molecules for the Early Diagnosis of Gastric MALT Lymphoma

Di Xiao[1†*], Qinghua Zou[2†], Le Meng[3], Yanli Xu[4], Huifang Zhang[1], Fanliang Meng[1], Lihua He[1], Jianzhong Zhang[1*]

Supplementary Table 1. IPA analysis results

Protein	Upstream molecules	Downstream molecules	Related diseases	Canonical Pathway	Biomarker	Expression and localization_Biofluid
>sp|P13667|GN=PDIA4	ATF6, XBP1	APOB, MTTP	Adenosquamous ovarian carcinoma, anaplastic carcinoma, liver carcinoma, thyroid cardinoma, benign thyroid nodule	No	No	No
>sp|P22626|GN=HNRNPA2B1	SLC18A3, TP53, VHL, HNRNPA2B1, CDKN1A, cevimeline, CHRM1, 5-fluorouracil, IL15, HRAS, ELOC, CD3, lith-O-Asp, mir-21, CST5	PKM, HNRNPA2B1, DDR1, SNAI2, ABCB1, H19, USH1C, UBASH3B, SVEP1, PHACTR2, MAST4, IDS, ADGRG2, FZD8, CEACAM7	Alzheimer disease, colorectal cancer, glioblastoma, glioblastoma cancer, glioma formation, inclusion body myopathy with early-onset Paget disease and frontotemporal dementia type 2, low grade astrocytoma, neoplasia, pilocytic astrocytoma, rheumatoid arthritis, vitamin D-resistant rickets	Telomere extension by telomerase, systemic lupus erythematosus signaling	No	Blood, Synovium/Synovial Fluid, Tears
>sp|P07711|GN=CTSL	PPARG	BCL2, Collagen type II, HPSE, MAP1LC3, BECN1	Pyoderma gangrenosum, Waldenstrm macroglobulinemia, abdominal aortic aneurysm, actinic keratosis, atopic dermatitis, cancer, colon cancer, diabetic nephropathy, epidermal hyperplasia, epithelial cancer, focal segmental glomerulosclerosis, folliculitis, hidradenitis suppurativa, leiomyosarcoma, lupus erythematosus, membranous glomerulonephritis, psoriasis, renal cancer, renal cell cancer, seborrheic keratosis, skin squamous cell	Autophagy; Phagosome Maturation	No	Blood, Plasma/Serum, Urine

			carcinoma, soft tissue sarcoma cancer, squamous cell skin cancer, ulcer			
>sp|Q08380|GN=LGALS3BP	IFNG, CDKN1A, IFNA2, TSH, doxorubicin, MAPK9, R5020, eflornithine, MDL 73811, MLX, epithelial cells, dexamethasone, tretinoin, RARA, CREBBP	TNF, IL6, IFNG, MMP3, IL12 (family), MMP14, FN1, IL12 (complex), MMP13, MHC CLASS I (family), PPIC, LGALS3BP, COLLAGEN type I	Adenocarcinoma, breast cancer, delayed hypersensitive reaction, epithelial cancer, psoriasis	No	prognosis-Ewing's sarcoma	Blood, Bronchoalveolar Lavage Fluid, Plasma/Serum, Sputum, Tears, Urine
>sp|Q9ULD2|GN=MTUS1	FOXA2, NKX2-1, CDX2, RASSF1, HUVEC cells, CD24, Ebna3c, D-glucose	ERK1/2, SNAI2, VIM, TP53, CDH1	Adenoid cystic carcinoma of salivary gland, epithelial cancer, organismal death, pancreatic neoplasia, pancreatic neoplasm, prostate cancer, salivary gland cancer	No	No	Blood, Plasma/Serum
>sp|P27824|GN=CANX	CZCL12, CASP1, Actin, CASP8, P-TEFb, TP73, ZDHHC6	BAX, NOX4, ABCA1, BCL2, DNM1L, FUNDC1, BCAP31	Alzheimer disease, Nonaka myopathy, gastric carcinoma, gastric epithelial cancer	Antigen Presentation Pathway; Lipid Antigen Presentation by CD1; Phagosome Maturation; Unfolded protein response	non-insulin-dependent diabetes mellitus	Blood, Cerebral Spinal Fluid
>sp|P67809|GN=YBX1	tretinoin, cisplatin, TGFB1, lipopolysaccharide, 5-fluorouracil, Akt, Calcineurin protein(s), TNF, INSR, CD 437, IRF4, peptidoglycan, ST1926, IL3, WT1	DNA endogenous promoter, DNA promoter, CDKN2A, ABCB1, COL1A2, CCL5, MVP, RNA polymerase II, HLA-DQB1, Havcr1, Ccl2, SNAI1, ABCC1, SV2C, PAPPA	Exencephaly, respiratory failure, cerebral hemorrhage, cyanosis, growth failure, edema, hypoplasia, sepsis, infection by HIV-1, neoplasia	Cancer Drug Resistance By Drug Efflux	No	Bronchoalveolar Lavage Fluid

Supplementary Table 2 High-affinity and intermediate-affinity MHC class I and II T cell epitopes of specific glycopeptides

Protein	Gene	Glycopeptide Sequence	MHC Class I T cell epitope		MHC Class II T cell epitope	
			Sequence (Positions) / Affinity	Alleles	Sequence (Positions) / Affinity	Alleles
P13667	PDIA4	MEPEEFDSDTLRE FVTAFKK	EEFDSDTLR (481-489)/ H	HLA-A*68:01	EPEEFDSDTLREFVT(489-503) /I	HLA-DRB3*01:01
			EEFDSDTLR (481-489) /I	HLA-A*33:01	EFDSDTLREFVTAFK(492-506) /I	HLA-DRB5*01:01, HLA-DRB3*01:01, HLA-DRB1*04:05
P22626	HNRNP A2B1	FGFVTFSSMAEVD AAMAARPHSIDGR V	SMAEVDAAMA(71-80) /H	HLA-B*35:01, HLA-A*02:03 , HLA-B*15:01	GFGFVTFSSMAEVDA(63-77) /H	HLA-DRB1*01:01
			SMAEVDAAMA(71-80)/ I	HLA-B*15:01 HLA-A*02:03 HLA-A*02:06 HLA-A*02:01 HLA-A*68:02	GFGFVTFSSMAEVDA(63-77) /I	HLA-DRB1*04:05, HLA-DRB1*01:01, HLA-DRB1*04:01, HLA-DRB1*09:01, HLA-DQA1*05:01/ DQB1*03:01
					FVTFSSMAEVDAAMA(66-80) /I	HLA-DRB1*01:01, HLA-DRB1*09:01
					GFVTFSSMAEVDAAM (65-79) /I	HLA-DQA1*05:01/ DQB1*03:01, HLA-DQA1*01:02/ DQB1*06:02
					FGFVTFSSMAEVDAA(64-78) /I	HLA-DQA1*05:01/ DQB1*03:01
					GFVTFSSMAEVDAAM(65-79) /I	HLA-DQA1*01:02/ DQB1*06:02
		GGNFGGSPGYGG GRG			GPGGGNFGGSPGYGG(250-264) /H	HLA-DQA1*05:01/ DQB1*03:01
					GNFGGSPGYGGGRGG(254-268) /I	HLA-DQA1*05:01/ DQB1*03:01, HLA-DRB1*01:01,
					GPGGGNFGGSPGYGG(250-264) /I	HLA-DRB1*01:01
					GGNFGGSPGYGGGRG(253-267) /I	HLA-DQA1*05:01/ DQB1*03:01
P07711	CTSL	KYSVANDTGFVDI PKQEKA			VANDTGFVDIPKQEK(219-233) /I	HLA-DRB1*11:01, HLA-DRB5*01:01
					NPKYSVANDTGFVDI(214-228) /I	HLA-DRB1*13:02
					KYNPKYSVANDTGFV(212-226) /I	HLA-DRB1*13:02
Q08380	LGALS 3BP	RALGFENATQALG RA			LGFENATQALGRAAF(65-79) /I	HLA-DRB1*01:01, HLA-DQA1*05:01/ DQB1*03:01, HLA-DQA1*01:02/ DQB1*06:02
					VCRALGFENATQALG(61-75) /I	HLA-DQA1*05:01/ DQB1*03:01
					VCRALGFENATQALG(61-75) /I	HLA-DRB1*04:01
					VCRALGFENATQALG(61-75) /I	HLA-DRB1*01:01
Q9ULD2	MTUS1	KVGPPVSCLRR			IAAPKAKVGPPVSCL(722-736) /I	HLA-DQA1*05:01/ DQB1*03:01
					VGPPVSCLRRNSDNR(729-743) /I	HLA-DRB1*11:01
					GPPVSCLRRNSDNRN(730-744) /I	HLA-DRB1*13:02
					PPVSCLRRNSDNRNP(731-745) /I	HLA-DRB1*11:01

T cell epitopes were classified based on their binding affinity for human major histocompatibility complex (MHC) alleles using the half-maximal inhibitory concentration of a biological substance (IC_{50}) as the unit of measure. MHC class I T cell epitope prediction was performed using the artificial neural network (ANN) method, and MHC class II T cell epitope prediction was performed using the consensus method, a combination of the average relative binding matrix method and the stabilization matrix alignment method (SMM-align). H: high-affinity binding, IC_{50} of <50 nM; I: ntermediate-affinity binding, IC_{50} of <500 nM; L: low-affinity binding, IC_{50} of <5000 Mm. A lower IC_{50} is indicative of a higher binding affinity to host MHC alleles. The underlined residues are the smm_align_core sequence. The gray amino acids are glycosylation sites.

Supplementary Table 3 Conformational B cell epitopes of specific glycopeptides

Protein	Gene	Glycopeptide Sequence	Conformational B Cell Epitopes		
			Peptides	Score	PDB ID
P13667	PDIA4	MEPEEFDSDTLREFVTAFKK	PEEFDSDTLRE (490-500)	0.606	5wwf.2
P07711	CTSL	KYSVANDTGFVDIPKQEKA	QYVQDNGGLDSEESYPYEAT EESCKYNPKYSVANDTG (188-224)	0.681	1cs8.1
Q08380	LGALS3BP	RALGFENATQALGRA	LGFENA (65-70)	0.557	1by2.1

A template model of each protein was obtained by submitting protein sequences in FASTA format and modeling those sequences. ElliPro was used to predict conformational B cell epitopes from selected proteins using a modeled 3D structure template for each protein. The default values (a minimum score of 0.5 and a maximum distance of 6 angstroms) were selected for 3D structure prediction. The gray amino acids are glycosylation sites.
