# Supplementary figures and images for: Glycopeptidomics Analysis of a Cell Line Model Revealing Pathogenesis and Potential Marker Molecules for the Early Diagnosis of Gastric MALT Lymphoma
Source: Front Cell Infect Microbiol. 2021 Aug 12;11:715454. doi: 10.3389/fcimb.2021.715454 (PMC8407071; doi:10.3389/fcimb.2021.715454)

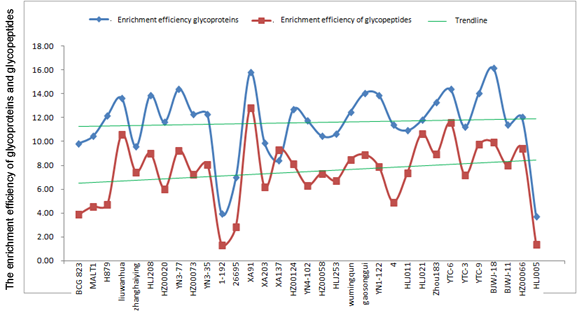

Supplement: Supplementary file 2 [file Image_1.tif]
